# Effects of the Cryptochrome CryB from *Rhodobacter sphaeroides* on Global Gene Expression in the Dark or Blue Light or in the Presence of Singlet Oxygen

**DOI:** 10.1371/journal.pone.0033791

**Published:** 2012-04-05

**Authors:** Sebastian Frühwirth, Kristin Teich, Gabriele Klug

**Affiliations:** Institut für Mikrobiologie und Molekularbiologie, Justus-Liebig-Universität, Giessen, Germany; North Carolina State University, United States of America

## Abstract

Several regulators are controlling the formation of the photosynthetic apparatus in the facultatively photosynthetic bacterium *Rhodobacter sphaeroides*. Among the proteins affecting photosynthesis gene expression is the blue light photoreceptor cryptochrome CryB. This study addresses the effect of CryB on global gene expression. The data reveal that CryB does not only influence photosynthesis gene expression but also genes for the non-photosynthetic energy metabolism like citric acid cycle and oxidative phosphorylation. In addition several genes involved in RNA processing and in transcriptional regulation are affected by a *cryB* deletion. Although CryB was shown to undergo a photocycle it does not only affect gene expression in response to blue light illumination but also in response to singlet oxygen stress conditions. While there is a large overlap in these responses, some CryB-dependent effects are specific for blue-light or photooxidative stress. In addition to protein-coding genes some genes for sRNAs show CryB-dependent expression. These findings give new insight into the function of bacterial cryptochromes and demonstrate for the first time a function in the oxidative stress response.

## Introduction

For photosynthetic organisms light can be beneficial as energy source but also harmful through generation of highly reactive singlet oxygen and the damaging effect of UV [1], [2]. In order to sense and to respond appropriately to changes in light quality or quantity microorganisms developed various regulatory mechanisms. Light-dependent responses can be mediated by signal pathways that depend on the photosynthetic electron transport or by photoreceptor-mediated signaling (reviews: [3], [4], [5], [6]).

*Rhodobacter sphaeroides* is a facultatively photosynthetic bacterium that forms photosynthetic complexes only at intermediate or low oxygen concentrations. Under aerobic conditions the PpsR protein represses transcription of photosynthesis genes and the bacteria perform aerobic respiration. When oxygen tension drops to intermediate levels (90 µM), the AppA protein binds to PpsR and allows transcription of photosynthesis genes in the dark. AppA can sense the redox status through a heme cofactor bound by its SCHIC domain [7], [8]. Under semiaerobic conditions blue light represses photosynthesis gene expression [9], [10]. Sensing of blue light by the BLUF domain of AppA (blue light sensing using FAD, [11]) releases PpsR that consequently represses photosynthesis genes [7]. At low oxygen tension (<3–4 µM) AppA is no longer binding to PpsR, no matter, whether light is present or not. Light even favors expression of photosynthesis genes under these conditions [10]. This stimulation depends on the photosynthetic electron transport, which through components of the respiratory chain signals to the PrrB-PrrA two component system [12]. PrrA is a major activator of photosynthesis genes in *R. sphaeroides*, when oxygen tension is low [13].

Homologs of the BLUF domain of AppA are found in many microorganisms and BLUF was established as a new class of photoreceptors [11], [14]. However, at least a second blue light photoreceptor, the cryptochrome CryB, affects expression of photosynthesis genes in *R. sphaeroides* [15]. Cryptochromes usually differ from the structurally related photolyases by their function in signal transduction but not in DNA repair [16]. In plants, cryptochromes regulate for example the cell elongation, photoperiodic flowering and stomatal opening and function through COP1 interaction [17], [18], [19]. Furthermore the regulation of the circadian clock in plants and animals are well characterized [20]. However, some members of the DASH family of cryptochrome [21] were shown to be associated with RNA and to photorepair thymine dimers in ssDNA [22], [23]. Therefore, Cry DASH proteins were supposed to be CPD (cyclobutane pyrimidine) -photolyases with specificity to CPD lesions in ssDNA [22].

The cryptochrome CryB of *R. sphaeroides* was shown to bind FAD and to undergo a typical photocycle [15]. The lack of CryB leads to some reduction of photoreactivation suggesting photolyase-like activity *in vivo* [24]. Recently the CryB crystal structure was solved and identified 6,7-dimethyl-8-ribityl-lumazine as antenna cofactor and a [4Fe-4S] cluster as third cofactor [25]. CryB is a member of a new class of Fe-S cluster containing proteins of the cryptochrome/photolyase family, which was recently predicted and named FeS-CPD (FeS Bacterial-Cryptochrome/Photolyases; [26]) and CryPro (proteobacterial cryptochromes), respectively [25]. *In vitro* a high affinity of CryB to single stranded DNA but no significant photorepair activity was observed. *cryB* is expressed under the control of an oxidative-stress dependent RpoH_II_ promoter and there was a significant effect of CryB on the amount of photosynthetic complexes and on the expression of photosynthesis genes [15].

In this study we have analyzed the effect of CryB on global gene expression by comparing the wild type strain to a mutant lacking CryB. This was performed for cultures grown at low oxygen tension (microaerobic conditions) and for cultures exposed to blue light under semiaerobic conditions or in the presence of methylene blue and light under aerobic conditions to generate high levels of singlet oxygen. The comparison of the different data sets allows to assess the specific role of CryB in the response to singlet oxygen and to blue light.

## Results

### Effect of CryB on the protein coding transcriptome at microaerobic, non-stress conditions

**Figure 1:**
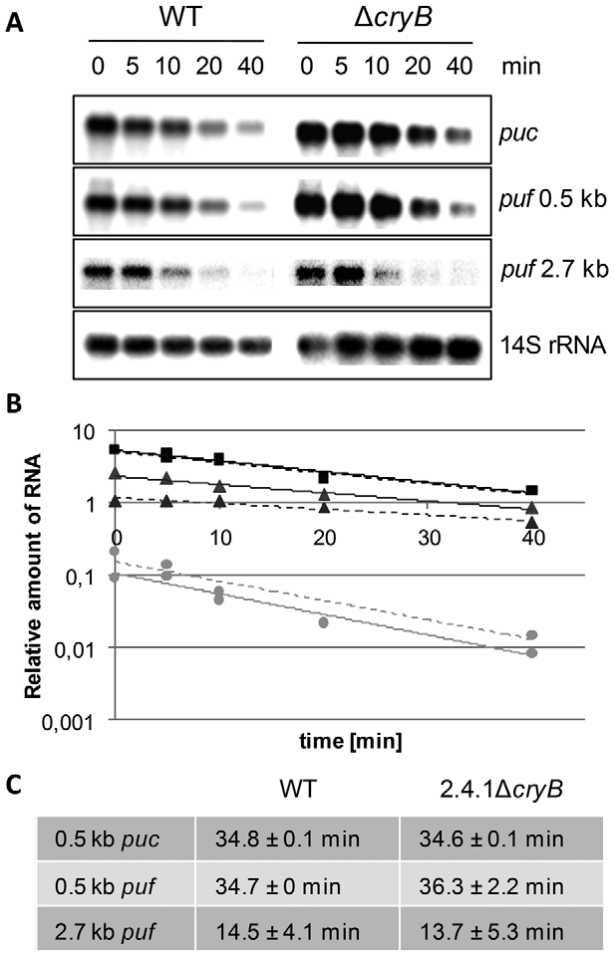
Stability determination of *puc* and *puf* mRNA. *puc* and *puf* mRNA encode structural proteins of the photosynthetic apparatus. (A) After addition of rifampicin at indicated time points, RNA was isolated and Northern blots were hybridized with *puc*- or *puf*-specific probes and re-hybridized with 14S rRNA-specific probes, serving as internal loading control. (B) Graphical analysis was used to determine the mRNA turn-over by normalizing *puc* and *puf* band intensities to the loading control and plotting against the time. Squares correspond to percentage of 0.5 kb *pucBA* mRNA, triangles display percentage of 0.5 kb *pufBA* mRNA and circles show percentage of 2.7 kb *pufBALMX* mRNA. Depicted are examples of *Rhodobacter sphaeroides* wild type (solid line) and the *cryB* deletion mutant (dashed line). (C) No significant changes in the turn-over of the mRNAs can be detected for *R. sphaeroides* wild type (WT) and the *cryB* deletion mutant.

The only obvious phenotypic effect of a deletion of the *cryB* gene from the chromosome of *R. sphaeroides* (2.4.1Δ*cryB*, formerly named 2.4.1Δ3077, [15], [24]) was a slightly lighter red color of the mutant compared to the wild type due to decreased amounts of pigment protein complexes. In a previous study we showed that CryB affects the *puf* and *puc* mRNA levels in *R. sphaeroides* [15]. The *puf* operon encodes proteins for the formation of the light-harvesting complex I and the reaction center. The *puc* operon encodes proteins for the formation of the light-harvesting complex II. The decreased levels of *puf* and *puc* mRNA could be due to decreased levels of transcription or to faster mRNA turn-over. To discriminate between these possibilities the half-lives of the 0.5 kb *pucBA*, the 0.5 kb *pufBA* and the 2.7 kb *pufBALMX* mRNAs in the wild type and in the mutant strain were determined under non-stressed, microaeobic conditions (Figure 1 A, B). Both short mRNA segments and the larger 2.7 kb transcript showed very similar half-lives of about 35 min and 14 min, respectively, in both strains implying that CryB rather affects the transcription of photosynthesis genes (Figure 1C).

Furthermore, it remained elusive, whether CryB specifically affects expression of photosynthesis genes or has a more global impact on gene expression. To investigate the impact of CryB on global gene expression we performed a comparative analysis of the transcriptomes of the wild type and the CryB mutant strain 2.4.1Δ*cryB*.

We applied a DNA microarray (GEO accession number GSE33556) based on the published genome sequence (NCBI project ID: 56) and including oligonucleotides directed against small RNAs (sRNAs), which were identified by (differential) RNA-Seq ([27], [28] and unpublished). Under non-stress conditions cultures were grown microaerobically (30 µM dissolved oxygen) in the dark. The complete set of results is displayed in Table S1. Within the set of appropriate A-values the expression level of 21% of these genes was higher in the mutant than in the wild type by a factor of 1.75 or more. Only 1.6% of all genes showed lower expression in the mutant compared to the wild type (by a factor of 0.6 or less).

Table 1 gives a brief overview on genes, grouped to functional categories, which are differentially expressed in the two strains under any of the tested conditions. Genes with different expression levels under microaerobic growth involve functional genes of the citric acid cycle, stress response, transcriptional regulators and other functions. However, the majority of differentially expressed genes are clustered in a wide group of transporters with various targets and in genes with unknown functions (Table S1).

**Table 1:** Expression of functionally related genes of *R. sphaeroides* 2.4.1Δ*cryB* compared to wild type under various conditions.

		Ratio of expression level mutant/wild type			
Category and RSP gene annotation	Gene	microaerobic non-stress conditions	semiaerobic 60 min. blue light	aerobic 20 min. ^1^O_2_	Description
**Photosynthesis**					
RSP_0256	*pufM*	0.98	**0.55**	0.72	Photosynthesis reaction center M subunit
RSP_0257	*pufL*	1.06	**0.49**	0.99	Photosynthesis reaction center L subunit
RSP_0291	*puhA*	1.08	0.60	0.71	Reaction center H protein
RSP_0314	*pucB*	1.40	(**0.52**)	1.32	LHII light harvesting B800/850 protein
RSP_1518	*prrA*	1.04	0.61	0.65	PrrA (RegA) response regulator
RSP_1520	*prrB*	(1.10)	(**0.37**)	(**0.39**)	Sensor histidine kinase PrrB (RegB)
RSP_6108	*pufB*	0.68	0.68	0.84	LHI light harvesting B875 subunit
**Citric acid cycle**					
RSP_4047	*pdhAa*	0.62	**0.42**	**0.40**	Pyruvate dehydrogenase E1 component
RSP_4049	*pdhAb*	**0.58**	**0.36**	**0.35**	Dihydrolipoamide acetyltransferase
RSP_4050	*pdhB*	**0.55**	**0.39**	**0.39**	Dihydrolipoamide acetyltransferase
**Oxidative phosphorylation**					
RSP_0296	*cycA*	0.90	0.70	**0.54**	Cytochrome c2
RSP_0693	*ccoP*	1.12	**0.59**	0.61	Cbb3-type cytochrome c oxidase CcoP subunit
RSP_0694	*ccoQ*	1.09	**0.56**	0.63	Cbb3-type cytochrome c oxidase CcoQ subunit
RSP_0994	*phaD*	(1.00)	**0.39**	0.61	NADH dehydrogenase subunit N
**Stress response**					
RSP_0601	*rpoH_II_*	(**1.91**)	(0.61)	0.65	RNA polymerase sigma factor
RSP_1092	*rpoE*	1.21	0.62	**0.58**	RNA polymerase sigma-70 factor
RSP_1546	*bfr*	1.23	0.68	**0.50**	Bacterioferritin
RSP_2293	*clpA*	(1.25)	(0.65)	**0.47**	Chaperonin clpA/B
RSP_2346		**0.57**	**0.35**	**0.43**	Cold-shock DNA-binding domain protein
RSP_2389		(1.42)	(0.64)	**0.43**	Putative glutathione peroxidase
RSP_2410	*rpoH_I_*	1.57	1.34	**0.55**	RNA polymerase sigma factor
**Transcriptional regulators**					
RSP_0402		(1.19)	(**0.47**)	(0.61)	Transcriptional regulator, TetR family
RSP_0847		**1.73**	**2.01**	**0.57**	Two component transcriptional regulator
RSP_0927	*lyrS*	(1.09)	(**0.37**)	(0.78)	Transcriptional regulator, LyrR family
RSP_3667		(0.70)	**0.45**	(0.64)	Transcriptional regulator, AraC family
**RNA processing/degradation**					
RSP_0224		(1.31)	(**0.49**)	(0.78)	ATP-dependent helicase
RSP_1112	*pnp*	0.70	0.62	0.67	Polyribonucleotide nucleotidyltransferase
RSP_1126	*rnr*	(1.17)	(**0.46**)	(0.61)	Exoribonuclease R
RSP_1971	*rnd*	(0.94)	**0.40**	(**0.60**)	Ribonuclease D
RSP_2131	*rne*	1.07	**0.60**	0.71	Ribonuclease E
RSP_2843	*hfq*	1.20	**0.59**	0.57	RNA-binding protein Hfq
**Others**					
RSP_0030		1.05	**0.39**	0.62	PAS sensor GGDEF/EAL domain
RSP_0905	*sitB*	1.20	0.75	**2.10**	ABC Mn+2/Fe+2 transporter, ATPase subunit
RSP_2877	*coxL*	1.11	0.71	**0.29**	Putative carbon monoxide dehydrogenase
RSP_3571	*znuA*	0.72	0.78	**2.97**	ABC zinc transporter
RSP_3539		**0.47**	**0.35**	**0.43**	Hemolysin-type calcium-binding region, RTX
RSP_3871	*modA*	**3.48**	(0.82)	(1.10)	ABC molybdate transporter
RSP_4157		**2.91**	(0.67)	(**0.53**)	Radical SAM superfamily protein
RSP_4158		**2.30**	(0.67)	(0.63)	Generic methyltransferase

Significant changes of the microarray data are in bold. Numbers in brackets failed to reach the set A-value criteria.

**Figure 2:**
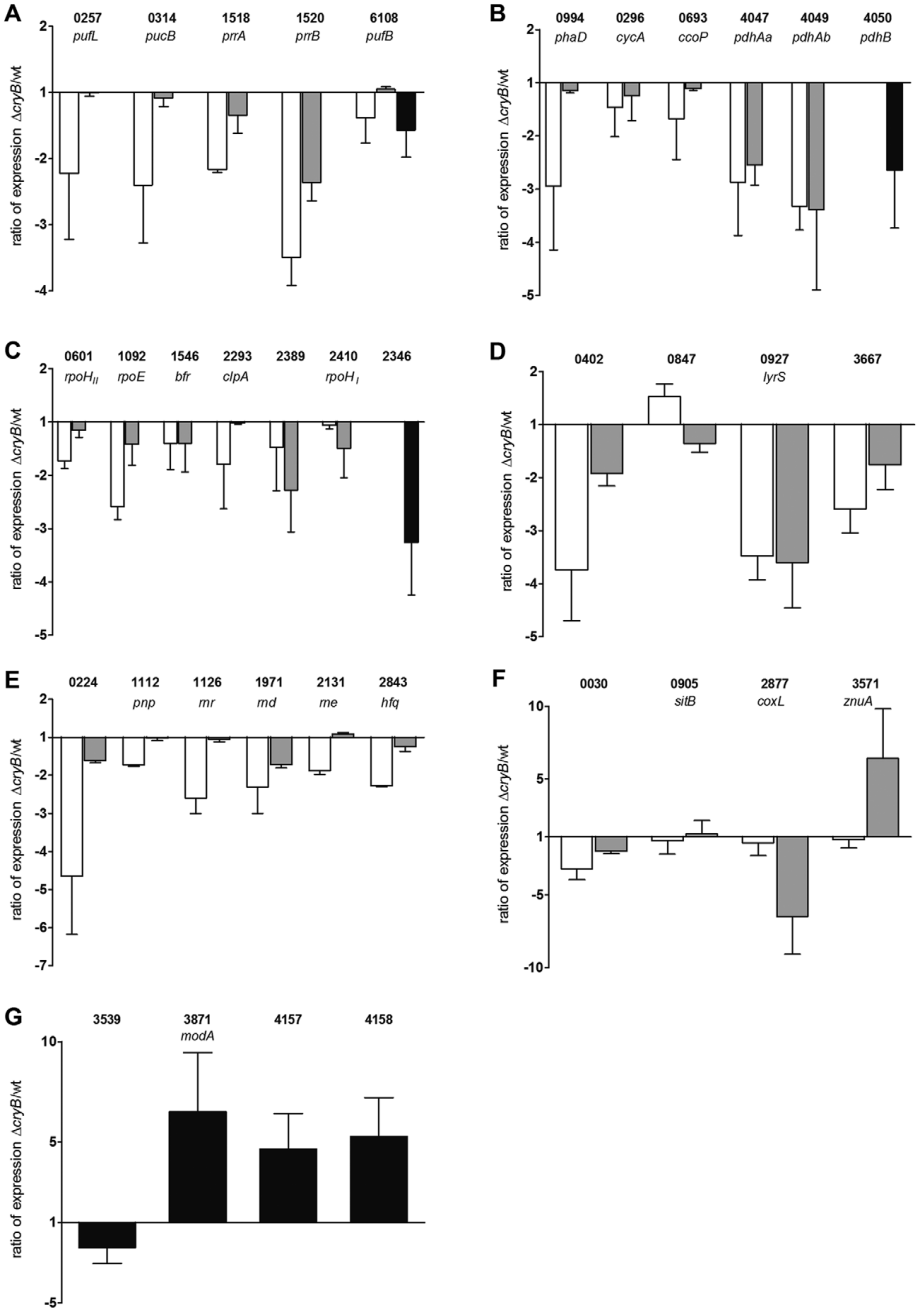
Expression ratio of selected genes as determined by real time RT-PCR. Cells were treated and total RNA was isolated and prepared for real time RT-PCR as described. Categories are clustered as described in Table 1, including genes involved in photosynthesis (A), citric acid cycle and oxydative phosphorylation (B), stress response (C), transcriptional regulators (D), RNA degradation and processing (E) and others (F, G). White bars indicate the expression ratio, comparing the *cryB* deletion mutant to the wild type, after 60 minutes semiaerobic blue light treatment. Grey bars depict the expression in 2.4.1Δ*cryB* after 20 minutes aerobic singlet oxygen treatment compared to the wild type treated in the same manner. Black bars show the expression ratio of the two strains after non-stressed, microaerobic growth. Numbers correspond to *R. sphaeroides* gene annotations. If gene name is missing, descriptions can be found in Table 1.

The microarray data obtained under microaerobic conditions were validated for selected genes by real time RT-PCR for the groups of energy metabolism (photosynthesis, citric acid cycle and oxidative phosphorylation, Figure 2A, B), stress response (Figure 2C) and for various other genes of different functions (Figure 2G). The real time data confirmed the changed ratios as observed by microarray analysis.

### Effect of CryB on the protein coding transcriptome after blue light illumination or in the presence of singlet oxygen

As shown previously [15] CryB binds FAD as a chromophore and undergoes a photocycle. Thus, it is likely to function as a photoreceptor and to mediate responses in a blue light dependent manner. Photoreceptors can sense very low light quantities, which do not lead to the generation of high levels of singlet oxygen. In *R. sphaeroides* AppA senses fluence rates of blue light as low as 0.2 µmol m^−2^ s^−1^, leading to decreased expression of photosynthesis genes [29]. Interestingly, the *cryB* gene itself is under the control of an RpoH_II_ dependent promoter [15]. RpoH_II_ was shown to target many genes in response to singlet oxygen [30] implying a role of CryB in this stress response.

To analyze singlet oxygen-dependent and blue light-dependent specific functions of CryB, transcriptome studies were performed in cultures containing methylene blue as artificial photosensitizer and illuminated with white light at high intensities (∼630 µmol m^−2^ s^−1^, contains about 7 µmol m^−2^ s^−1^ of blue light) for 20 min or in cultures illuminated with blue light of lower total fluence rate (∼20 µmol m^−2^ s^−1^ blue light).

**Figure 3:**
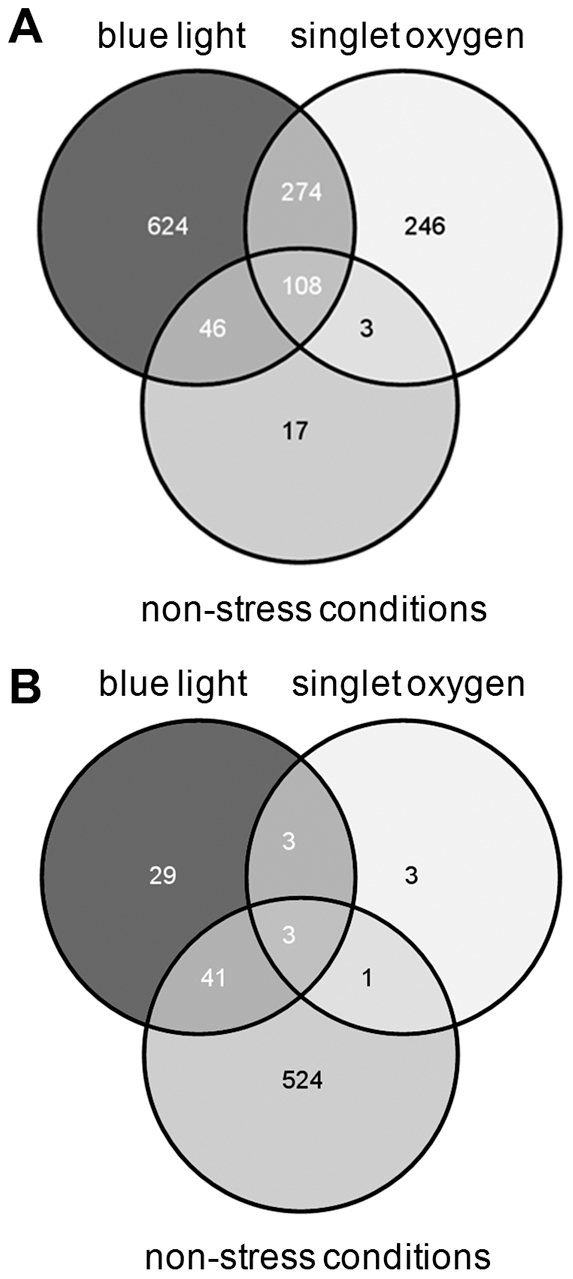
Venn diagram of all differentially expressed genes in 2.4.1Δ*cryB* compared to wild type. Depicted are genes with decreased (A) and increased expression (B) in 2.4F1Δ*cryB* under different conditions. Numbers correspond to protein coding genes and putative small RNAs that were significantly differentially expressed in the *cryB* deletion mutant compared to wild type. A complete list of the microarray data is shown in Table S1.

A Venn diagram summarizing differentially expressed genes in 2.4.1Δ*cryB* compared to the wild type under different conditions is depicted in Figure 3. Note that under blue light or singlet oxygen stress conditions most genes showed lower expression levels in the mutant (Figure 3A), while under non-stressed, microaerobic conditions most genes showed higher expression levels (Figure 3B, Table S1). Under blue light illumination the expression level of only 2.3% of all the genes that reached satisfying A-values was higher in the mutant by a factor of 1.75 or more compared to the wild type. About 39.5% showed lower expression in the mutant than in the wild type by a factor of 0.6 or less. In the presence of singlet oxygen the expression level of only 0.4% of all the genes that reached satisfying A-values was higher in the mutant by a factor of 1.75 or more compared to the wild type. 26% of the genes showed lower expression in the mutant than in the wild type by a factor of at least 0.6. The latter result implicates a role of CryB in the singlet oxygen response. Therefore the sensitivity of the 2.4.1Δ*cryB* mutant to exposure to methylene blue in the light was compared to that of the wild type by zone inhibition assays. We repeatedly observed slightly smaller inhibition zones for the mutant indicating increased resistance to singlet oxygen (data not shown). The small differences for the two strains were however statistically not significant.

A main defense factor against hydrogen peroxide is catalase, and the *catA* gene of *R. sphaeroides* shows strong increase in response to this ROS [31], [32]. *CatA* expression was not significantly affected by CryB. Thus not all genes, which are strongly regulated by ROS or have important functions in ROS protection, show CryB dependent expression.

Table 1 gives an overview on the genes that are differentially expressed in the CryB mutant compared to the wild type under the selected growth conditions. Strong differences could be seen in the category of photosynthesis, where functional regulators such as PrrB/PrrA [13], [33] but also genes encoding structural parts of the photosynthetic apparatus such as *puc* and *puf* showed lower expression in 2.4.1Δ*cryB* compared to the wild type under blue light exposure. Under ^1^O_2_ exposure within the category photosynthesis only the PrrB/PrrA regulator system seemed to be affected by the deletion of *cryB*. Similar expression ratios between the two strains in blue light and singlet oxygen treated cells were observed for genes of the citric acid cycle and oxidative phosphorylation, for stress responses, and for some transcriptional regulators. Here, most genes showed similarly lower expression in the CryB mutant compared to wild type under both conditions. However, slight differences between blue light and singlet oxygen conditions were observed in the group of RNA processing and degradation, where most genes showed far lower expression ratios under blue light exposure and for some of the transcriptional regulators, first and foremost *ompR* (RSP_0847) (Table 1 and Figure 2).

As illustrated by the Venn diagrams (Figure 3) there was a big overlap of singlet oxygen and blue light effects on CryB-dependent expression. Nevertheless, most of the CryB-dependent genes showed lower expression in the mutant under blue light illumination. Genes, which showed higher expression in the mutant, were mostly affected under non-stressed, microaerobic growth. These data demonstrate that the effect of blue light on CryB-dependent expression is specific and not just a consequence of low levels of singlet oxygen, which are produced during illumination.

### Effect of CryB on the expression of selected genes in response to blue light illumination or ^1^O_2_ treatment

To validate the microarray data we performed real time RT-PCR for some selected genes. Figure 2 shows the expression of these genes in the *cryB* mutant compared to the wild type. Blue light treated experiments (white bars) were compared to singlet oxygen stress (grey bars) and non-stressed, microaerobic experiments (black bars) in the different groups as classified in Table 1.

As predicted by the microarray data, all genes of the photosynthesis group showed less expression in the CryB mutant compared to the wild type under blue light exposure (Figure 2A, white bars), while this was only the case for *prrA* (RSP_1518) and *prrB* (RSP_1520) for singlet oxygen treated cells (Figure 2A, grey bars). In the case of other energy metabolism pathways (citric acid cycle, oxidative phosphorylation, Figure 2B) and the stress response (Figure 2C) all tested genes showed a similar expression ratio when comparing the CryB mutant to the wild type as they were predicted by the microarray (Table 1). Note that in the real time RT-PCR data, *rpoH_II_* (RSP_0601), *rpoE* (RSP_1092) and *clpA* (RSP_2293) showed significantly lower expression ratios under blue light exposure (Figure 2C, white bars) compared to ^1^O_2_ treatment (grey bars), while it was vice versa for *rpoH_I_* (RSP_2410).

**Figure 4:**
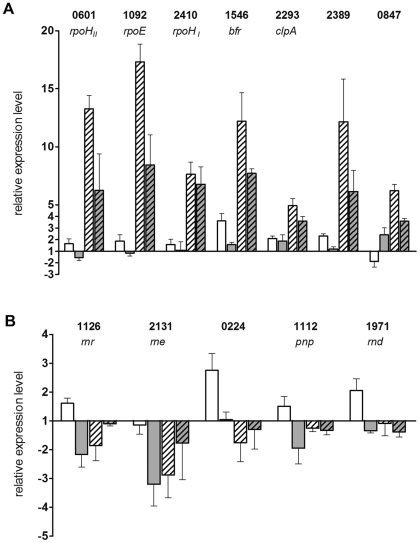
Relative expression levels of selected genes as determined by real time RT-PCR. Real time RT-PCR was performed for selected genes of the stress response (A) and genes of RNA processing and degradation (B). Cells were treated by blue light illumination or ^1^O_2_ exposure and RNA was isolated as described. To visualize gene induction or repression RNA was also isolated of untreated cells. White bars depict the *R. sphaeroides* wild type after 60 min blue light treatment compared to untreated cells. Grey bars show the results for the *cryB* deletion mutant under the same conditions. White, striped bars correspond to wild type treated by 20 min ^1^O_2_ compared to unstressed cells. Grey, striped bars show the results for the *cryB* deletion under photooxidative stress. Gene annotations are the same as listed in Table 1 or Figure 2.

To follow the expression change of selected genes for stress responses in response to external stimuli, RNA was isolated at 0 and 20 min of photooxidative stress or 0 and 60 min blue light illumination and real time RT-PCR was performed (Figure 4A). The results reveal that these genes show only very weak response to blue light (white and grey bars) but strong response to singlet oxygen in both strains (white, striped and grey, striped bars). With the exception of RSP_2410 encoding *rpoH_II_* and *clpA* (RSP_2293) all selected genes showed significantly stronger responses in the wild type than in the CryB mutant.

By real time RT-PCR the microarray data could also be validated for the group of transcriptional regulators (Figure 2D) and RNA processing and degradation (Figure 2E). Strikingly, a significant difference in the expression ratios between blue light and ^1^O_2_ treated cells was confirmed for the two component transcriptional regulator RSP_0847. The synthesis rate of the RSP_0847 gene product, a putative OmpR homologue, was strongly increased upon ^1^O_2_ exposure in the wild type [34]. Its levels were reduced in the *rpoH_I_* and the *rpoH_II_* mutants and not detectable in the *rpoH*_I_/_II_ double mutant [30]. Therefore, it seems likely that the lower expression levels of *rpoH_II_* in the *cryB* mutant under ^1^O_2_ conditions (Figure 4A) consequently lead to a lower expression ratio of *ompR* when comparing the CryB mutant to wild type (Figure 2D). However, the expression of *ompR* is higher in 2.4.1Δ*cryB* compared to the wild type upon blue light illumination (Figure 2D), although *rpoH_II_* showed lower expression in the mutant compared to wild type under these conditions (Figure 2C). To better understand the effect of CryB on *ompR* expression, real time RT-PCR was performed at 0 and 20 min after onset of singlet oxygen stress. As expected, *ompR* transcript levels increased in the wild type (factors of about 6, Figure 4A, white, striped bar). An increase of the *ompR* transcript level in response to singlet oxygen was also observed in 2.4.1Δ*cryB*, but this increase (factor of about 3) was less pronounced compared to the wild type (Figure 4A, grey, striped bar). After 60 min of blue light illumination *ompR* expression was repressed in the wild type (Figure 4A, white bar) while it is induced in the CryB mutant (grey bar).

Several genes of the RNA processing and degradation group showed low expression ratios under blue light exposure (Figure 2E, white bars). After singlet oxygen treatment only the ATP-dependent helicase (RSP_0224) and *rnd* (RSP_1971) showed significantly reduced expression ratios when comparing the *cryB* mutant to wild type (Figure 2E, grey bars). The change in expression levels in response to singlet oxygen or blue light was quantified by real time RT-PCR for these genes. As seen in Figure 4B singlet oxygen caused no significant change in the expression level for most genes (white, striped and grey, striped bars). However, with the exception of *rne* (RSP_2131) all these selected genes were slightly induced by blue light illumination in the wild type (Figure 4B, white bars). In the *cryB* mutant all genes, except of the ATP-dependent helicase (RSP_0224), were significantly repressed under blue light illumination (Figure 4B, grey bars).

Gene expression changes in the group of unclassified, other genes were also confirmed by real time RT-PCR (Figure 2F). The altered expression ratios under ^1^O_2_ exposure but not under blue light illumination were validated for *coxL* (RSP_2877) and *znuA* (RSP_3571) but not for *sitB* (RSP_0905).

### Effect of CryB on the expression of sRNAs under different conditions

As shown previously [27] the levels of several sRNAs of *R. sphaeroides* were affected under various stress conditions. The microarray used in this study contained 144 oligonucleotides derived from intergenic regions, including the sRNAs RSs0680a, 0682, 0019 and 2461. All these sRNAs are either induced or processed in response to oxidative stress [27]. For the latter three a reduced expression level in 2.4.1Δ*cryB* compared to wild type was detected under blue light illumination and ^1^O_2_ conditions in this study (Table 2). For RSs0680a a significantly increased ratio of expression levels was observed when comparing the CryB mutant to the wild type after ^1^O_2_ treatment but not after blue light illumination. When cultures were grown under non-stress conditions a higher expression level of RSs0680a was also observed for the mutant. Note that here the cells were grown to a higher OD_660_ of 0.8 and that the oxygen tension further decreased to 30 µM compared to blue light illumination experiments (90 µM). Surprisingly, RSs2461, which is a processing product of a co-transcript with RSP_0847 (putative *ompR* homologue, [27]), showed lower expression levels in 2.4.1Δ*cryB* under blue light and ^1^O_2_ conditions (Table 2). For *ompR* higher expression levels in the mutant were observed for blue light illumination and lower levels under singlet oxygen stress in the microarrays as well as by real time RT PCR (Table 1).

**Table 2:** Expression of small RNAs in *R. sphaeroides* 2.4.1Δ*cryB* compared to wild type under various conditions.

	Ratio of expression level mutant/wild type			
RSs no.	microaerobic non-stress conditions	semiaerobic 60 min. blue light	aerobic 20 min. ^1^O_2_	Description from Berghoff et al., 2009
RSs0680a	**1.94**	1.26	**1.84**	Co-transcription with RSP_6037 from an RpoH_I/II_ promoter, induced by ^1^O_2_ and O^−^_2_ exposure.
RSs0682	0.81	**0.25**	**0.39**	Processing after ^1^O_2_ exposure in an Hfq-dependent manner.
RSs0019	1.68	0.78	**0.54**	RpoE-dependent induction under ^1^O_2_ exposure.
RSs2461	1.08	**0.43**	**0.50**	Co-transcription with RSP_0847 from an RpoH_I/II_ promoter, induced after ^1^O_2_ and O^−^_2_ exposure.

Significant changes of the microarray data are in bold.

**Figure 5:**
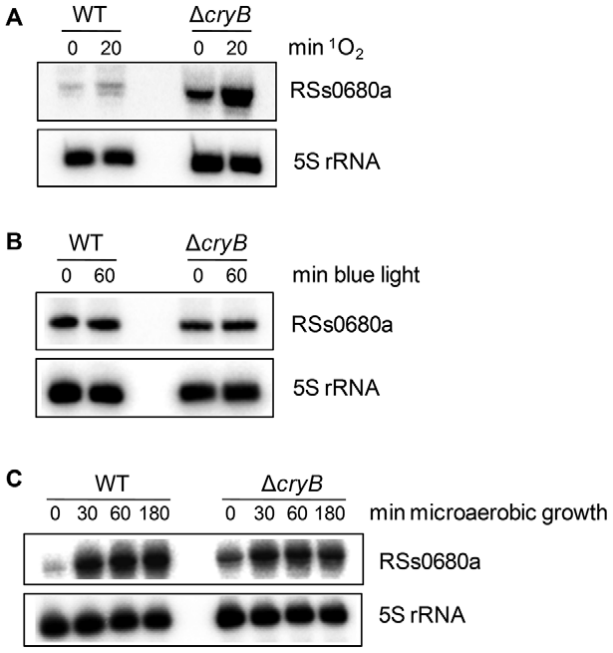
Northern blot analysis of the small RNA RSs0680a under different conditions. RSs0680a expression is increased in 2.4.1Δ*cryB* under singlet oxygen (A) but not under blue light conditions (B) or microareobic growth (C). Experiments were performed as described and 12 µg of total RNA seperated on 10% polyacrylamid gels containing 7 M urea. 5′-labeled oligodeoxynucleotide probes were used for detection of RSs0680a and 5S rRNA, which served as internal loading control. RSs0680a expression is induced approximately 2–3 times after 20 minutes of ^1^O_2_ exposure in *Rhodobacter sphaeroides* wild type (WT) and the *cryB* deletion mutant compared to unstressed cells (0 min). The levels of the sRNA after 0 and 20 minutes ^1^O_2_ treatment, respectively are increased approximately 5–6 times in 2.4.1Δ*cryB* compared to WT. No significant differences in expression can be detected under blue light treatment. Oxygen shift experiments show that RSs0680a is more abundant in 2.4.1Δ*cryB* under aerobic conditions (time point 0 min). After shifting cultures to non-stressed, microaerobic conditions no significant difference in RSs0680a expression is detected for both strains.

The microarray data revealed that the sRNA RSs0680a was higher expressed in 2.4.1Δ*cryB* compared to wild type under singlet oxygen and microaerobic growth, while expression under blue light was similar. To validate these data and to follow the expression change of RSs0680a in response to ^1^O_2_ and decreased oxygen tension Northern blot analysis was performed. Figure 5A shows the expression levels of RSs0680a after 0 and 20 minutes of ^1^O_2_ treatment. Clearly, a higher initial level was detected in the CryB mutant already before the onset of ^1^O_2_ stress. The sRNA level in 2.4.1Δ*cryB* was approximately 4 times higher at time point 0 minutes compared to the wild type and 5 times higher after 20 minutes of ^1^O_2_. The wild type showed an increase of the RSs0680a level of approximately 2.2 when comparing the signal after 20 minutes ^1^O_2_ to time point 0 minutes. The RSs0680a level in the CryB mutant was increased to approximately 2.7 after 20 minutes ^1^O_2_ treatment. As predicted by the microarray data, no significant difference could be detected for RSs0680a levels under blue light conditions between the mutant and the wild type (Figure 5B). After aerobic, non-stressed growth a higher expression of RSs0680a was determined for the CryB mutant compared to the wild type (Figure 5C). However, a significant increase of the small RNA in the mutant compared to wild type was not observed when shifting the cultures to non-stressed, microaerobic conditions (Figure 5C). RSs0680a is co-transcribed together with the protein-coding gene RSP_6037 from an RpoH_I/II_ promoter [27]. Surprisingly, the gene RSP_6037 itself did not show altered expression under ^1^O_2_ exposure but showed slightly higher expression ratios under non-stress, microaerobic conditions in the microarray data (Table S1).

The expression levels of RSs0019, which is induced under ^1^O_2_ exposure, did not change when comparing the CryB deletion to wild type cultures under these conditions (data not shown), although this was predicted by the microarray data.

## Discussion

While an effect of the cryptochrome CryB on photosynthesis gene expression was already demonstrated [15] it was not known, whether this protein also affects other genes of *R. sphaeroides* and whether the effect on gene expression is influenced by external stimuli. The data presented in this study reveal that CryB also affects many other genes besides photosynthesis genes and that both blue light and singlet oxygen influence this effect. Thus, it rather functions as a global regulator than as specific regulator for photosynthesis. In the past a transcriptome analysis was performed for a mutant of S*ynechocystis* sp. PCC 6803 lacking the sII1629 gene, which was suggested to function as cryptochrome [35]. About two-fold lower expression of eight genes in the sII1629 mutant compared to the wild type was observed. This difference was, however, statistically significant only for two genes of unknown function [21]. Thus the sII1629 gene product of *Synechocystis* has much less effect on global gene expression than CryB of *R. sphaeroides*.

There is a large overlap of the CryB-dependent effects of blue light and singlet oxygen. This is expected since the generation of singlet oxygen requires illumination of the cultures, which will induce the CryB photocycle. However, for some genes specific effects are observed. For example photosynthesis genes and genes for RNA metabolism show stronger CryB-dependent effects in response to blue light than in response to singlet oxygen. This is in agreement with the expected function of CryB as a photoreceptor and excludes the possibility that the response to blue light is caused by low levels of singlet oxygen under blue light illumination. This also implies that these blue-light specific effects need a higher fluence rate of blue light than present in the white light used to generate singlet oxygen. However, only very few genes are affected through CryB by singlet oxygen but not by blue light (Figure 2F). Our data also reveal a CryB-dependent effect on gene expression in the dark. Remarkably, different sets of genes are affected in the dark or under blue light or stress conditions. Light-independent effects were also described for other cryptochromes. E. g. mammalian Cry1 and Cry2 were shown to act as light-independent components of the circadian clock [36], [37]. Blue light-independent functions were also observed for Arabidopsis cryptochromes [38], [39].

Altogether our observations suggest that CryB can affect gene expression by different signal chains. While its interaction to some downstream partners may require the light dependent excitation of the FAD, other interactions may be influenced by the redox changes of the CryB iron-sulfur cluster or by both. Future experiments with variants of CryB lacking one of these cofactors will elucidate the role of the CryB cofactors in different responses. CryB may be the second protein of *R. sphaeroides*, besides AppA, which can sense light and redox signals through different cofactors and integrate these signals.

### Overlap of the CryB and other regulons

Among the genes affected by CryB are genes for structural components of the photosynthetic apparatus and regulators of photosynthesis genes (Hendrischk et al., 2009 and this study). Since we did not detect direct binding of CryB to promoter regions of photosynthesis genes [15], it is conceivable that CryB affects gene expression through interaction with other regulatory proteins. A main role in the regulation of photosynthesis genes has the PpsR/AppA system. The PpsR regulon comprises mostly photosynthesis genes [40], [41]. Furthermore, photosynthesis genes are controlled by the PrrB/PrrA two component system, which activates transcription under low oxygen tension [13]. The PrrA regulon comprises also genes with no function in photosynthesis and its regulation [40].

The microarray data predict very slight but consistent changes of all PpsR-regulated genes in the CryB mutant compared to wild type (Table S1). This was validated for some genes by real time RT-PCR. This implies that the AppA/PpsR system may be involved in CryB-dependent signaling, but that CryB has only a small, modulating effect on AppA/PpsR.

PrrA binding motifs were found upstream of several photosynthesis genes (*puf*BALMX, *puh*A, *puc*BAC, [40]). As the expression ratio of *prrA* between mutant and wild type is reduced under blue light and also slightly under ^1^O_2_ conditions (Table 1 and Figure 2A) this could contribute to the reduced expression of the photosynthesis genes in the *cryB* deletion mutant compared to the wild type. Furthermore, genes of a CO dehydrogenase operon (RSP_2879-76) harbor a PrrA binding site and are differentially expressed in 2.4.1Δ*cryB* compared to wild type (Table S1). Interestingly, this operon shows a lower expression ratio under ^1^O_2_ conditions. This could be validated for RSP_2879 and RSP_2877 by real time RT-PCR. On protein level a reduced abundance of CoxL (RSP_2877) and CoxM (RSP_2876) was detected in an *rpoH*_I/II_ deletion mutant [30].

**Figure 6:**
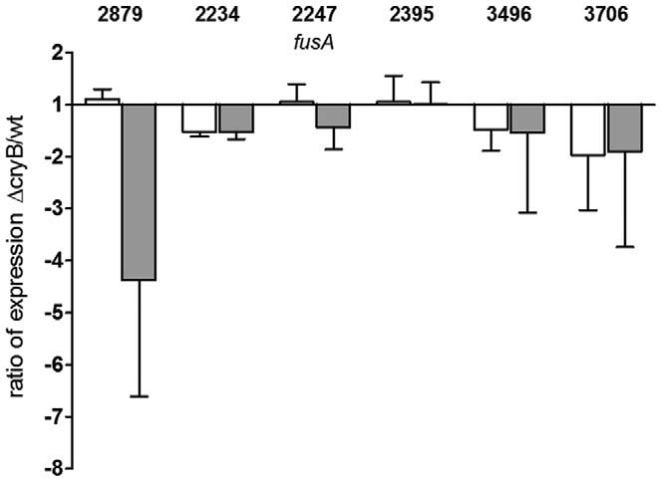
Effects of CryB on PrrA-regulated genes. Real time RT-PCR experiments of selected genes with predicted PrrA binding sites show that CryB has no general effect on genes regulated by the PrrB/PrrA system under the tested conditions. White bars correspond to the ratio of expression of the CryB mutant compared to wild type after blue light illumination. Grey bars depict the expression ratios after ^1^O_2_ treatment. Numbers correspond to *R. sphaeroides* gene annotations. RSP_2879, hypothetical protein, *cox* operon; RSP_2234, predicted DNA-binding protein; RSP_2395, BCCP, cytochrome c peroxidase; RSP_3496, zinc carboxypeptidase A metalloprotease; RSP_3706, hypothetical protein.

Although there is a partial overlap between some photosynthesis genes that possess a PrrA binding motif and the CryB regulon, Figure 6 clearly shows that only very few tested genes of the PrrA regulon are significantly altered in expression by a deletion of *cryB*. Others show insignificant tendencies or are clearly not affected by the deletion under blue light or ^1^O_2_ conditions, respectively. We conclude that CryB does not influence gene expression through the PrrA/PrrB system.

Since expression of *catA* is not significantly affected by CryB and *catA* expression strongly depends on OxyR [31], we can also exclude signaling of CryB through the OxyR transcriptional regulator.

### CryB, a general regulator of the energy metabolism and stress adaptation

The largest set of differentially expressed genes in 2.4.1Δ*cryB* compared to wild type is clustered in the functional groups photosynthesis, citric acid cycle and oxidative phosphorylation (Table 1). According to COG categories approximately 60% of all genes belonging to the category oxidative phosphorylation were affected by CryB under blue light or singlet oxygen conditions. Together with genes of the citric acid cycle, approximately 20% of the total energy metabolism genes were affected. Slight effects for those genes were also visible under dark, non-stress conditions. It is striking that the expression ratio of several cytochromes is reduced as they play major roles in respiration and photosynthesis. An effect of a cryptochrome on energy and stress-related gene products was also described in tomato plants [42].

Compared to other functional categories genes affected by CryB are over-represented in the category energy metabolism. CryB affected only 8% of all photosynthesis genes, 4% of the transcriptional regulators, and 9% of genes of the category RNA processing/degradation, respectively. It is conceivable that CryB has an effect on the general energy metabolism, antagonizing the down-regulation of genes for energy metabolism under stress conditions. To avoid formation of (photo) oxidative stress, a limitation of the photosynthetic apparatus, or reactive oxygen species forming components of the respiratory chain, seems reasonable [1]. CryB could counterbalance this repression. Growth curves show that the CryB mutant exhibit delayed growth and also does not reach as high cell densities as the wild type (Figure S1). This would be in agreement with a function of CryB in maintaining the energy production on reasonable levels.

### Effect of CryB on RNA processing and sRNA expression

It was shown previously that the deletion of *cryB* resulted in some reduced abundance of *puc* and *puf* mRNA [15]. To discriminate between a CryB effect on transcription or on mRNA turn-over we determined the *puc* and *puf* mRNA half-lives by Northern blot analyses (Figure 1). No significant differences in mRNA turn-over were detected for the CryB mutant or *R. sphaeroides* wild type. Nevertheless, several RNases showed reduced expression in 2.4.1Δ*cryB* compared to wild type (Table 1, Figure 2E, 4B and Table S1). RNase E and RNase III are the major endonucleases in gram-negative bacteria and initiate the decay of many mRNAs [43]. PNPase is involved in polyadenylation and like RNase R functions as 3′ to 5′exoribonuclease. Unlike the situation in *E. coli*, in *R. sphaeroides* PNPase is not a main component of the RNA degrading degradosome complex, which is organized by RNase E [44]. Due to the important functions of these proteins it can be expected that changes in expression of the corresponding genes can affect stability of other mRNAs and thus indirectly transcript levels. However, we did not observe any significant change in the half-life for the 2.7 kb *pufBALMX* transcript, which is known to be degraded in an RNase E dependent manner [45], [46].

This study reveals that CryB does not only affect protein-coding genes but also genes for sRNAs. The binding of sRNAs to their targets often leads to faster or slower degradation of both RNAs [47]. Since several genes for RNases (Table 1) and also the gene for Hfq, a RNA chaperone, involved in mRNA degradation [48], [49] and sRNA-mRNA interaction [48], [50], are reduced in their expression in strain 2.4.1Δ*cryB* it is conceivable that CryB affects sRNA expression levels post-transcriptionally. Such post-transcriptional effects can also explain the different effects CryB has on certain sRNAs and the protein-coding part of their precursors as observed for the sRNA RSs2461, which is co-transcribed together with the *ompR* gene and for RSs0680a, which is co-transcribed together with the protein-coding gene RSP_6037. In the case of the small regulatory RNA RSs0680a higher expression levels can be seen in the *cryB* deletion mutant under ^1^O_2_ exposure (Figure 5A). So far a regulatory role cannot be addressed to RSs0680a, although it is clearly co-transcribed together with RSP_6037 from an RpoH_I/II_ promoter [27].

### Conclusions

This study reveals that CryB of *R. sphaeroides* affects expression of numerous genes with different biological functions. While the expression level of some genes is increased in a CryB mutant, the expression level of others is decreased. For most genes CryB only affects blue light-dependent expression but very few are also regulated in a ^1^O_2_-dependent manner. This suggests that CryB affects expression of individual genes by different downstream signaling pathway. Considering the large number of CryB-dependent genes it is likely that the effect on some genes is indirect. Some of the CryB-dependently expressed genes are transcriptional regulators or affect RNA stability and can therefore indirectly influence expression levels of other genes.

## Materials and Methods

### Bacterial strains, growth conditions and blue light or ^1^O_2_ stress experiments

*Rhodobacter sphaeroides* was grown at 32°C in malate minimal salt medium [51]. For non-stressed, microaerobic growth experiments, cells were allowed to grow in Erlenmeyer flasks under continuous shaking at 140 r.p.m. (dissolved oxygen concentration of approximately 30 µM). Samples for RNA isolation were collected when the cultures reached an OD_660 nm_ of 0.8, exactly. Blue light experiments were performed as described elsewhere [10], [52]. Cultures inoculated to an OD_660 nm_ of 0.15 were kept under semiaerobic conditions (approximately 90 µM dissolved oxygen) by varying the rotation speed of the shaker. After one doubling time blue light (λ_max_ = 400 nm with 20 µmol m^−2^ s^−1^ at the culture level) was passed through a band pass filter (BG12 Schott, light transmitted from 300 nm to 500 nm, [10]). Samples for RNA isolation were collected 60 min after the onset of blue light irradiation. For singlet oxygen experiments cultures were grown under aerobic conditions by gassing with air (dissolved oxygen of approximately 180 µM) in flat glass bottles. Cultures were grown in the dark to an OD_660 nm_ of 0.4. Generation of ^1^O_2_ was induced by adding methylene blue (final concentration of 0.2 µM) and illuminating the aerobic cultures with high intensities of white light (800 W m^−2^, 630 µmol m^−2^ s^−1^, [1]). Samples for RNA isolation were taken 20 min after the onset of white light illumination.

### RNA preparation

Total RNA for microarray and Northern blot analyses was isolated by using the hot phenol method [53]. After DNase I treatment the RNA was purified by using mixtures of phenol-chloroform-isoamylalcohol (25∶24∶1) and chloroform-isoamylacohol (24∶1). For microarray analysis the RNA was further purified by RNeasy®MinElute™ spin columns (Qiagen) following the manufacturer's instructions. For real time RT-PCR experiments total RNA was isolated by Total RNA isolation reagent (TRIR, ABGene) according to the manufacturer's specifications.

### Zone inhibition assays

For the measurement of sensitivity to ^1^O_2_ exponential phase grown cultures were diluted to an OD_660 nm_ of 0.05. 0.5 ml were diluted into 5 ml prewarmed top agar (0.8%) and layered on minimal salt medium plates. Filter paper discs were placed on top of the plates and 5 µl of 10 mM methylene blue (Sigma-Aldrich) applied to the discs. Plates were incubated for 48 h at 32°C under a fluorescent tube (Spectralux Plus, NL36 W/860 daylight) or wrapped in aluminum foil as dark control.

### Northern blot analysis and half-life experiments

Northern blot analysis of small RNAs was performed as described by Berghoff et al. [27]. Probes for detection of RSs_0680a and RSs_2430 are listed in Table S3, published by Berghoff et al. [27]. For half-life experiments microaerobically grown over night cultures of *Rhodobacter sphaeroides* were diluted to an OD_660_ of 0.8. A control sample was taken before adding 0.2 mg/ml final concentration of rifampicin. RNA was isolated as described above. Northern blot analysis for determination of *puc* and *puf* mRNA half-lives was performed as described by Braatsch et al. [10]. mRNA half-lives were calculated out of three independent repeats from corresponding graphical analyses.

### Oxygen shift experiments

400 ml of *R. sphaeroides* culture were grown aerobically in a 2 l beaked flask at 32°C over night. Exponential phase grown cultures were diluted to an OD_660 nm_ of 0.2 and allowed to grow aerobically to an OD_660 nm_ of 0.4. Aerobic samples were taken for RNA isolation and the culture was shifted to a 500 ml flask for microaerobic growth, immediately. Microaerobic samples were taken at indicated time-points.

### Real time RT-PCR

Primers used for analyzing the expression of different target genes are listed in Table S2. Real time RT-PCR was performed following the specifications of the one-step RT-PCR kit (Qiagen) with a final concentration of 4 ng/µl total RNA. Sybr green I (Sigma-Aldrich) was added in a final dilution of 1∶50,000 to the master mixture. Real time RT-PCR data were normalized against *rpoZ* (omega subunit of RNA polymerase). Further conditions were followed as described previously [1], [34].

### Microarray analysis

The microarray contains probes against 4,304 protein-coding genes, 79 rRNA and tRNA genes, and 144 intergenic regions. Three antisense probes with a length of 60 nt were designed for each gene or intergenic region, when possible. Microarray construction was performed following the instructions of Agilent (www.chem.agilent.com). The ULS™ Fluorescent Labeling Kit for Agilent arrays (Kreatech) was used for RNA labeling and fragmentation. The RNA of three independent experiments of *Rhodobacter sphaeroides* wild type and the *cryB* deletion mutant were pooled and hybridized to one array. A total of three arrays (non-stressed, microaerobic growth) or two arrays (blue light and singlet oxygen experiments) including nine or six biological repeats, respectively, were used. Gene chip hybridization and scanning were performed according to the specifications from Agilent. Multiarray analysis was performed with the Bioconductor package Limma for R [54], [55]. Background correction and normalization (locally weighted scatterplot smoothing) were performed as described previously [56], [57]. A-values were calculated to express the reliability of gene expression. An A-value of ≥9.5 (non-stressed, microaerobic growth) or 12 (blue light and singlet oxygen experiments) was considered satisfying. Cut-off values of 1.75 for increased expression and 0.6 for reduced expression were used to show significant changes in expression levels of the *cryB* deletion mutant compared to the control treatment (*Rhodobacter sphaeroides* wild type 2.4.1). Microarray data was published on Gene Expression Omnibus (http://www.ncbi.nlm.nih.gov/geo/), GEO accession number GSE33556.

## Supporting Information

Figure S1 **Growth curves of** ***Rhodobacter sphaeroides*** **wild type, 2.4.1Δ** ***cryB*** **and the complementing mutant 2.4.1Δ** ***cryB*** **pRK** ***cryB*** **.** Fresh overnight cultures were diluted to an OD_660 nm_ of 0.2 with a total volume of 75 ml in a 100 ml flask. For OD measurements, samples of 1 ml were taken and the flasks refilled with 32°C pre-warmed malate minimal salt medium, immediately. OD_660 nm_ was measured in 1 h time points and plotted in logarithmic scale. *R. sphaeroides* 2.4.1 wild type is shown as black curve, the *cryB* deletion mutant is depicted as grey curve and the *cryB* mutant complementing the defect from the plasmid pRK*cryB* is shown as grey, dashed line.
(TIF)

Table S1 ***Rhodobacter sphaeroides*** **transcriptome data under various conditions.** The whole transcriptome dataset is added in the supporting material. Genes are sorted by their corresponding gene annotations (RSP number). A-values in green reached the internally set A-value criteria, following MA blots, normalized by loess. Ratios were calculated between the *cryB* deletion mutant and *R. sphaeroides* wild type. Arrows indicate an increased or reduced gene expression in the *cryB* deletion mutant compared to the wild type (under corresponding conditions). Mean values were calculated from two (blue light, singlet oxygen) or three arrays (non-stressed) including each RNA pools of three independet, biological repeats. More than one value for the same gene indicates different probes on the chip (see probe name). Singlet oxygen, blue light and non-stress experiments were performed as described in material and methods. Microarray data is published on Gene Expression Omnibus (http://www.ncbi.nlm.nih.gov/geo/), GEO accession number GSE33556.
(XLS)

Table S2 **Real time RT-PCR primer sequences.** The primer list contains the corresponding *R. sphaeroides* gene annotations and gene names. Primers are listed including their annealing temperature, primer efficiency and oligonucleotide sequences (5′–3′).
(PDF)
