# Supplementary figures and images for: Effects of the Cryptochrome CryB from Rhodobacter sphaeroides on Global Gene Expression in the Dark or Blue Light or in the Presence of Singlet Oxygen
Source: PLoS One. 2012 Apr 5;7(4):e33791. doi: 10.1371/journal.pone.0033791 (PMC3320616; doi:10.1371/journal.pone.0033791)

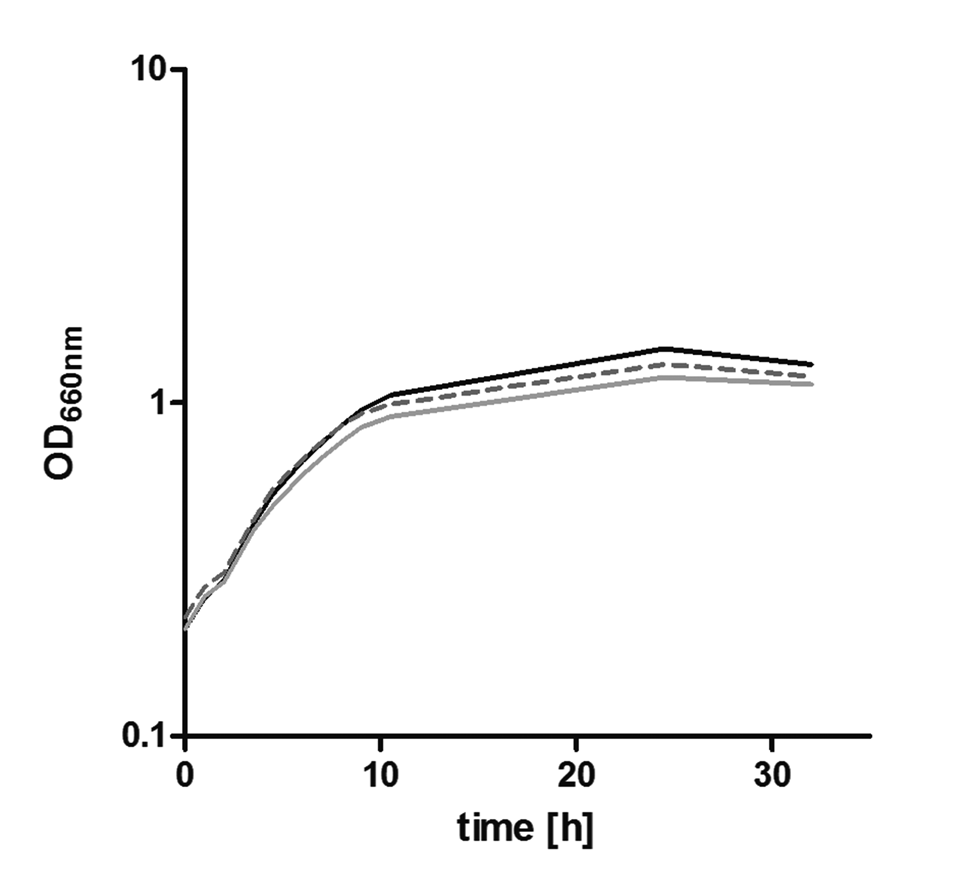

Supplement: Figure S1 — Growth curves of Rhodobacter sphaeroides wild type, 2.4.1Δ cryB and the complementing mutant 2.4.1Δ cryB pRK cryB . Fresh overnight cultures were diluted to an OD660 nm of 0.2 with a total volume of 75 ml in a 100 ml flask. For OD measurements, samples of 1 ml were taken and the flasks refilled with 32°C pre-warmed malate minimal salt medium, immediately. OD660 nm was measured in 1 h time points and plotted in logarithmic scale. R. sphaeroides 2.4.1 wild type is shown as black curve, the cryB deletion mutant is depicted as grey curve and the cryB mutant complementing the defect from the plasmid pRKcryB is shown as grey, dashed line. (TIF) [file pone.0033791.s001.tif]
