# Supplementary material for: Effects of the Cryptochrome CryB from Rhodobacter sphaeroides on Global Gene Expression in the Dark or Blue Light or in the Presence of Singlet Oxygen
Source: PLoS One. 2012 Apr 5;7(4):e33791. doi: 10.1371/journal.pone.0033791 (PMC3320616; doi:10.1371/journal.pone.0033791)
Supplement: Table S2 — Real time RT-PCR primer sequences. The primer list contains the corresponding R. sphaeroides gene annotations and gene names. Primers are listed including their annealing temperature, primer efficiency and oligonucleotide sequences (5′–3′). (PDF) [file pone.0033791.s003.pdf]

Table S2. Real time RT-PCR primer sequences. The primer list contains the corresponding *R. sphaeroides* gene annotations and gene names. Primers are listed including their annealing temperature, primer efficiency and oligonucleotide sequences (5' – 3').

primer pair	gene	efficiency	annealing temp. [°C]	sequence (5' – 3') forward / reverse
RSP_1669	*rpoZ*	2.02	59	ATCGCGGAAGAGACCCAGAG / GAGCAGCGCCATCTGATCCT
RSP_0030		2.02	56	AGGAGATCATGCGCTACG / GATCAGCGGCTGGTAGAA
RSP_0224		1.74	56	TACGCTTGGCGACATGGA / GTTGCGGATTGGCTGCTT
RSP_0257	*pufL*	2.34	56	ACACCTACGGCAACTTCC / ATCGAGTAGCCGACCAGA
RSP_0296	*cycA*	1.92	56	TCAACCAGTGCCAGACCT / GTCCGCTTCCTTCTTCAG
RSP_0314	*pucBA*	2.01	56	CGAAGCCGAAGAAGTTCA / TTCACCACGAGCCAGATT
RSP_0402		1.85	56	GGTTCCACATCGGCTATC / CCTCAAGACGCTCCTCAT
RSP_0601	$rpoH_{II}$	2.09	56	GCCGATGAACGACCTGAT / AAGAACAGCGCCTTCTGG
RSP_0693	*ccoP*	2.11	56	TCCGAACCTGCTGGATGA / TTGTCGGCGAAGATGGTG
RSP_0694	*ccoQ*	1.89	56	GTCTTCTTCGAGGTTCTG / GGTTGTAGGCCACATACT
RSP_0847		1.87	56	GCGCACGAACAATCAGAG / TGGCGCAACCTGTAGATG
RSP_0905	*sitB*	1.97	56	CTATGTGCCGCAATCCGA / AACACGCGCTTCCTCTGA
RSP_0927	*lyrS*	2.03	56	GTTCCTGCTCGACTATCC / GTGAGGTCGTCGATCTTC
RSP_0994	*phaD*	2.02	56	GTCTTCTTCGAGGTTCTG / GGTTGTAGGCCACATACT
RSP_1092	*rpoE*	2.04	56	GTCTGGCAGAAGGCTCAT / GTTCTCCTGCTGCATCTC
RSP_1112	*pnp*	2.09	56	GACTATGCCGCGCTCTAT / GCTCGTCTCCGAGATGAT
RSP_1126	*rnr*	2.00	56	ACCGGCTGATCGAGGAAT / GTTCAGATGCGCGGTCTT
RSP_1173	*dnaK*	1.94	56	CGACATGCGGATCGTCAA / CCGGTCCATGCTGATGAA
RSP_1518	*prrA*	2.11	56	GCTTCTCGTGGACGATGA / AGGTCCACCACTGCATAG
RSP_1520	*prrB*	1.97	56	AGTTCCAGGCGCTGATGA / AGGATGCCGATGACGATG
RSP_1546	*bfr*	2.09	56	CAGCGCATCATCTTCCTC / ATAGTCGCGCACCTTCTC
RSP_1971	*rnd*	2.06	56	ACGAATGCCGAAGAGCTG / TTCCGACTTGGCCTTGAG
RSP_2131	*rne*	2.01	56	TGCGGTTGTCAACTTCCT / TCGCGGTCTTGAACTTCT
RSP_2234		1.89	56	ATGCCTCGACGCTCTATG / CACCTCGAAGCCGTAGAA
RSP_2247	*fusA*	1.93	56	TATACCTGCGGCAAGACG / CGACAGTTCGGTGCTCAT
RSP_2293	*clpA*	1.88	56	CTACGACGCGGTCAACTT / CGGCAGAGAACCTGAATG
RSP_2346		1.90	56	CGGCTTCGCCAATGTCTTCG / AGGCCGGTTGCTGATGCTGA
RSP_2389		2.24	56	CCAACTTCGACCTGACAC / GTCACGGTCGATCAGAAC
RSP_2395		2.01	56	GAAGGACAACCGCATCAC / TGGCAGGAATAGCAGGAG
RSP_2410	$rpoH_{I}$	2.00	56	GATCGCCAAGGATCT / CTGGTCGCTGTCTTCA
RSP_2843	*hfq*	2.02	56	AGGACGCCTTTCTGAACC / TCGCCCTCGTAGAGATTG
RSP_2877	*coxL*	1.91	56	TACCAGCAGGCCAAGGAT / GGCGGTTGTTCACCAGTT
RSP_2879		2.04	56	TGTCACGCTGTCGGACAT / AACCGCTCGAAGAACTGG
RSP_3496		1.97	56	TGGACGCTCCACAATCTC / CCTGTGCAACACCGGATA
RSP_3539		1.78	56	TGAACGCGCAGGAAGGCAAC / ATCACGCGCCAGATTGACCG
RSP_3571	*znuA*	2.03	56	GATCACGGCGAACATGAG / GGCATCGTGGAAGACGTA
RSP_3667		1.89	56	TGACGCTGACCGATGTGA / TGCGCCATCCGTATGAGT
RSP_3706		1.96	56	TCGATCTCGGCATCTACC / CACATCCTTGCGCAGAAC
RSP_3871	*modA*	2.06	56	CAACAGCGTCACGATCTCCT / GACCTCGTCCATCCAGTTCA
RSP_4047	*pdhAa*	2.03	56	ACAAGCGCATCACCTCCT / TGTCGTTGCCGAGATAGC
RSP_4049	*pdhAb*	2.23	56	CCAGCACAGCCAGGATTA / GATGCCGAAGGAGACGAT
RSP_4050	*pdhB*	2.11	56	GCACCGCACGAATATCAG / CGAGAGCGTCATCGACAT
RSP_4157		1.86	56	ATAAGATCCGCGAGCTGAAG / ATCACGCTGCTAATCGTCAC
RSP_4158		1.99	56	GCGGCTATTCGGCGAAGATG / TCAAGCACCGCGTCCTTCAG
RSP_6108	*pufBA*	2.00	62	CAGGCGCAGGAATTGCACTC / GAACCACGGACGCCAGATGT
